# FibroTest for Evaluating Fibrosis in Non-Alcoholic Fatty Liver Disease Patients: A Systematic Review and Meta-Analysis

**DOI:** 10.3390/jcm10112415

**Published:** 2021-05-29

**Authors:** Yasaman Vali, Jenny Lee, Jérôme Boursier, René Spijker, Joanne Verheij, M. Julia Brosnan, Quentin M. Anstee, Patrick M. Bossuyt, Mohammad Hadi Zafarmand, on behalf of the LITMUS Systematic Review Team

**Affiliations:** 1 Department of Epidemiology and Data Science, Amsterdam UMC, University of Amsterdam, 1105 AZ Amsterdam, The Netherlands; j.a.lee@amsterdamumc.nl (J.L.); p.m.bossuyt@amsterdamumc.nl (P.M.B.); m.h.zafarmand@amsterdamumc.nl (M.H.Z.); 2 Hepato-Gastroenterology Department, Angers University Hospital, 49933 Angers, France; JeBoursier@chu-angers.fr; 3 HIFIH Laboratory, UPRES EA3859, Angers University, 49035 Angers, France; 4 Medical Library AMC, Amsterdam UMC, University of Amsterdam, 1105 AZ Amsterdam, The Netherlands; r.spijker@amsterdamumc.nl; 5 Cochrane Netherlands, Julius Center for Health Sciences and Primary Care, University Medical Center Utrecht, 3584 CX Utrecht, The Netherlands; 6 Department of Pathology, Amsterdam UMC, University of Amsterdam, 1105 AZ Amsterdam, The Netherlands; j.verheij@amsterdamumc.nl; 7 Internal Medicine Research Unit, Pfizer Inc., Cambridge, MA 02139, USA; Julia.brosnan@pfizer.com; 8 The Newcastle Liver Research Group, Translational & Clinical Research Institute, Faculty of Medical Sciences, Newcastle University, Newcastle upon Tyne NE1 7RU, UK; quentin.anstee@newcastle.ac.uk; 9 Newcastle NIHR Biomedical Research Centre, Newcastle upon Tyne Hospitals NHS Foundation Trust, Newcastle upon Tyne NE1 7RU, UK

**Keywords:** non-alcoholic fatty liver disease, liver fibrosis, diagnostic tests, FibroTest, cirrhosis

## Abstract

(1) Background: FibroTest™ is a multi-marker panel, suggested by guidelines as one of the surrogate markers with acceptable performance for detecting fibrosis in patients with non-alcoholic fatty liver disease (NAFLD). A number of studies evaluating this test have been published after publication of the guidelines. This study aims to produce summary estimates of FibroTest™ diagnostic accuracy. (2) Methods: Five databases were searched for studies that evaluated FibroTest™ against liver biopsy as the reference standard in NAFLD patients. Two authors independently screened the references, extracted data, and assessed the quality of included studies. Meta-analyses of the accuracy in detecting different levels of fibrosis were performed using the bivariate random-effects model and the linear mixed-effects multiple thresholds model. (3) Results: From ten included studies, seven were eligible for inclusion in our meta-analysis. Five studies were included in the meta-analysis of FibroTest™ in detecting advanced fibrosis and five in significant fibrosis, resulting in an AUC of 0.77 for both target conditions. The meta-analysis of three studies resulted in an AUC of 0.69 in detecting any fibrosis, while analysis of three other studies showed higher accuracy in cirrhosis (AUC: 0.92). (4) Conclusions: Our meta-analysis showed acceptable performance (AUC > 0.80) of FibroTest™ only in detecting cirrhosis. We observed more limited performance of the test in detecting significant and advanced fibrosis in NAFLD patients. Further primary studies with high methodological quality are required to validate the reliability of the test for detecting different fibrosis levels and to compare the performance of the test in different settings.

## 1. Introduction

Non-alcoholic fatty liver disease (NAFLD) is a potentially progressive disorder, representing a wide spectrum of disease from simple steatosis and different degrees of fibrosis to non-alcoholic steatohepatitis (NASH), and eventually cirrhosis. With a global prevalence of approximately 25%, NAFLD is now the leading cause of chronic liver disease worldwide and a growing challenge to public health [1,2]. NAFLD is the manifestation of metabolic syndrome in the liver and is strongly associated with obesity, especially combined with insulin resistance [3,4,5]. This association explains the high prevalence of NAFLD in the obese population, which affects a significant number of patients with a body mass index (BMI) over 30 kg/m^2^ globally [5,6].

There is evidence that liver fibrosis stage is the most potent prognostic factor for NAFLD patients. Any progress in the fibrosis stage (from less than F2 to F3 and F4) is associated with a higher risk of long-term outcomes and an increase in liver-related mortality [7,8,9,10,11]. Hence, early identification of NAFLD patients with advanced fibrosis (F3/4) is recommended by international guidelines [12,13,14] and is a key area of interest for clinical trial recruitment [15].

Liver biopsy is currently recommended as the reference standard for detecting NASH and hepatic fibrosis [16]. However, its suitability for diagnosis in clinical practice or in drug development has been questioned, because of the costly and invasive nature of this procedure, and the risk of potentially severe or even fatal complications [17,18,19]. The limitations of the liver biopsy have fueled the development of non-invasive NAFLD biomarkers.

FibroTest™ (FibroSURE in the US) is a panel of biochemical markers that originally was developed for the assessment of bridging fibrosis in patients with hepatitis C (HCV) [20]. Later, it has also been evaluated for other chronic liver diseases, including hepatitis B (HBV) [21,22], alcoholic liver disease (ALD) [23,24], and NAFLD [25].

The EASL-EASD-EASO Clinical Practice Guidelines recommend FibroTest™ as a non-invasive biomarker with acceptable diagnostic accuracy, as defined by an area under the receiver operating characteristic curve (AUC) > 0.80 for detecting fibrosis and NAFLD progression [16]. This guideline, published in 2016, referred to only two studies for this recommendation [25,26]. By now, more studies that evaluated the accuracy of this biomarker in NAFLD patients have been published, with varying levels of performance. It is unclear whether the basis for the recommendation in the guideline still corresponds to the current body of evidence.

To provide more precise summary estimates of clinical performance and to explore likely sources of variability in the reported test accuracy, we performed a comprehensive systematic review and meta-analysis of studies of the performance of FibroTest™ in detecting any fibrosis (F1–F4), significant fibrosis (F2–F4), advanced fibrosis (F3–F4), or cirrhosis (F4) in NAFLD patients.

## 2. Materials and Methods

This study was conducted as part of LITMUS (Liver Investigation: Testing Marker Utility in Steatohepatitis), a large multi-center project funded by the European Union IMI2 scheme. This project aimed to evaluate a set of biomarkers for detecting NASH and fibrosis in NAFLD patients. The protocol of the full systematic review is available in PROSPERO (CRD42018106821). The report of this study was prepared using the PRISMA-DTA statement [27] (see Supplementary Table S1).

### 2.1. Search Methods

Using a comprehensive and sensitive search strategy, developed in close collaboration with our search specialist (R.S.), we searched five electronic databases: Medline (via OVID), EMBASE (via OVID), PubMed, Science Citation Index, and CENTRAL (The Cochrane Library). Our search strategy looked for words in the title/abstract or across the record and in the medical subject heading (MeSH). This strategy was initially run against the databases in August 2018, and it was updated in May and December 2019 and 2020. We limited our search to human subjects but applied no further restrictions based on either year or language (See Supplementary Table S2 for the Medline search strategy).

In addition, we manually scrutinized the reference lists of related systematic reviews and of the articles reporting all included studies to identify additional studies. We also contacted academic and industry partners within the LITMUS consortium to identify any potentially missed publications.

### 2.2. Inclusion and Exclusion Criteria

#### 2.2.1. Index Biomarker

The index test for this review is FibroTest™ (Biopredictive Paris), also known as FibroSURE (LabCorp, Burlington, NC, USA), the brand name for FibroTest in the USA. This panel consists of serum α2-macroglobulin, apolipoprotein A1, haptoglobin, total bilirubin, and gamma-glutamyltranspeptidase (GGT), adjusted for age and gender. It provides a quantitative estimate of liver fibrosis, ranging from 0 to 1, with 0 referring to F0 and 0.74 to F4 [28].

ActiTest, another test from Biopredictive Paris, which includes the same components plus alanine-aminotransferase (ALT), is used for assessment of necro-inflammatory activity; this test, however, was not included in this systematic review [29].

#### 2.2.2. Target Condition

The target conditions of interest are NASH (with or without fibrosis) and different stages of fibrosis: any fibrosis (F1–F4), significant fibrosis (F2–F4), advanced fibrosis (F3–F4), and cirrhosis (F4). We considered the five-point scoring system (F0–F4), developed by NASH Clinical Research Network (CRN) staging system, or any other scoring systems [30] for inclusion. The scores reported by studies that had used alternative scoring systems, other than NASH CRN, were converted into the corresponding NASH CRN equivalent. More information about the different scoring systems for liver fibrosis [30,31,32,33,34,35,36,37] and characterizing changes in NAFLD progression [30,38,39,40,41] is provided in Supplementary Tables S3 and S4.

#### 2.2.3. Study Design and Participants

Potentially eligible were studies, reported in full-text articles or as conference abstracts, that had enrolled adult patients (≥18 years) with biopsy-proven or suspected NAFLD and reported FibroTest™ results (the index test) with paired liver histology data as a reference standard. We did not include studies that had recruited patients with other chronic liver conditions (e.g., viral hepatitis) or decompensated cirrhosis, and we only considered studies in patients with mixed etiologies if the performance of FibroTest™ in NAFLD patients was reported separately. We excluded studies without enough data to calculate diagnostic accuracy estimates from our meta-analysis.

### 2.3. Data Collection and Analysis

#### 2.3.1. Selection of Studies

We removed duplicate records of our initial search results. All remaining titles were screened by one reviewer (Y.V.), while a second reviewer independently screened 10% of the titles. The abstracts and full-text reports of all potentially eligible studies were independently screened by two authors (Y.V. and J.L.). A decision on inclusion was reached in discussions with the third reviewer (M.H.Z.) in case of any disagreement. The Rayyan software (https://rayyan.qcri.org, accessed from August 2018 to January 2021) was used in the screening phase of this study.

#### 2.3.2. Data Extraction

The following data were extracted by one author (Y.V./J.L.) and cross-checked by the other author (J.L./Y.V.): study group characteristics, index test and reference test features, and number of true and false positives and true and false negatives for constructing classification tables. The corresponding authors of studies that did not report sufficient data for reconstructing contingency tables were contacted.

#### 2.3.3. Assessment of Methodological Quality

We used the Quality Assessment of Diagnostic Accuracy Studies tool (QUADAS-2) to assess the risk of bias and concerns about applicability in included studies [42]. The risk of bias of each of the four main domains of the review-specific QUADAS-2 tool was evaluated by two authors (Y.V. and J.L.) independently, and a judgment of ’low’, ’high’, or ’unclear’ risk was assigned to each study.

#### 2.3.4. Statistical Analysis

Classification tables were extracted or reconstructed for expressing the diagnostic accuracy of FibroTest™, for each pre-defined target condition. Study-specific estimates of sensitivity, specificity, and corresponding 95% confidence intervals (CI) were generated and graphically illustrated in forest plots. Accuracy data were extracted for reported thresholds. Variability was assessed based on visual assessment of these forest plots and ROC curves.

In this systematic review, we did not attempt to formally evaluate publication bias since funnel plot asymmetry, the usual test in reviews, cannot discriminate between publication bias and other sources of asymmetry in systematic reviews of test accuracy studies [43].

Since studies could report a single threshold or multiple threshold values for different target conditions, we applied two different meta-analytical methods, depending on the number of reported threshold values. The bivariate random-effects model (mada package in R) was applied to calculate summary estimates of sensitivity, specificity, and predictive values of studies that reported only one threshold value. Since this method is based on evaluation of a single threshold, calculation of the predictive values in alternative threshold values is not possible.

When sensitivity and specificity have been reported for multiple threshold values by a single study, we used a linear mixed-effects model (diagmeta package in R). This multiple thresholds model enabled us to express summary estimates of sensitivity and specificity at different cut-points as well as the calculation of the predictive values, depending on the prevalence of the target condition of interest [44,45]. The threshold values are calculated based on maximizing Youden’s J statistic (also called Youden’s index): the sum of sensitivity and specificity minus 1. In our calculations of predictive values, a range of prevalences was used, based on the observed prevalence in our included primary studies. We further evaluated FibroTest thresholds required to achieve pre-specified high values of sensitivity and specificity. For each of the meta-analyses, SROC curves were constructed to represent the overall diagnostic accuracy of the index test in detecting the corresponding target condition.

We calculated 95% confidence intervals and 95% prediction intervals around estimates, where appropriate. The confidence interval around the mean represents the range of values that are still plausible, given the included studies. A prediction interval also includes the between-study heterogeneity and refers to plausible values that a future single primary accuracy study of the same index test, for the same target condition, may generate.

A minimally acceptable performance level of 0.80 (for sensitivity, specificity, and AUC) was predefined for FibroTest™, which would exceed the performance of other NAFLD-related fibrosis screening and diagnostic biomarkers [16]. R for Windows (Version 3.6.0; R Foundation for Statistical Computing, Vienna, Austria) was used in all analyses.

We investigated the influence of type of scoring method for fibrosis staging in a sensitivity analysis, by removing a study that used METAVIR criteria from meta-analysis for advanced fibrosis [26]. We also assessed the effect of the scoring method type on the meta-analysis for significant fibrosis, by comparing the results before and after adding the single study using the METAVIR system.

## 3. Results

### 3.1. Findings

#### 3.1.1. Search Results

**Figure 1:**
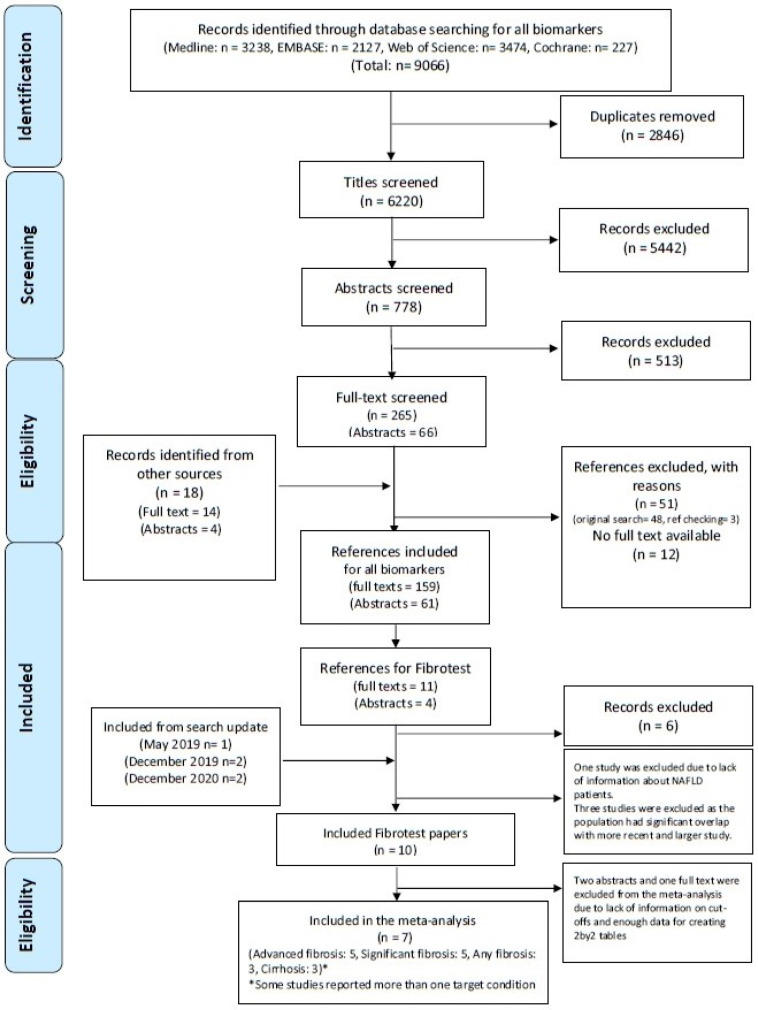
PRISMA flow diagram of primary studies.

After removing duplicates from the 9066 references found by the initial search, we screened 6220 titles, 778 abstracts, and 265 full-text reports. We found 18 studies after searching other sources. Fifteen potentially eligible studies reporting on the accuracy of FibroTest™ in NAFLD patients were evaluated in the first search, while we found five more eligible studies after updating the search. In total, ten studies met all of our inclusion criteria and were included in our systematic review, and seven of them could be included in our meta-analysis (Figure 1). See Supplementary Table S5 for reasons of exclusions.

#### 3.1.2. Study Characteristics

The characteristics of the ten studies that met our inclusion criteria are summarized in Table 1. The mean or median age of the participants included in these studies ranged from 42.2 to 57 years. Six studies had included NAFLD patients with a mean BMI ≥ 25, and one study had recruited morbidly obese participants (BMI ≥ 35) [26]. The percentage of diabetic patients was reported by seven studies, and it ranged from 23% to 100%.

Of the seven studies included in our meta-analysis, five studies had reported on the performance of FibroTest™ in detecting advanced fibrosis [25,26,46,48,51] and significant fibrosis [25,48,49,51,52], while performance for the other target conditions (any fibrosis [26,48,49] and cirrhosis [48,51,52]) was reported by only three studies. There was a wide range in the proportion of study participants with the respective target conditions: 41–91% for any fibrosis levels, 27–68% for significant fibrosis, 11–41% for advanced fibrosis, and 10–14% for cirrhosis.

The included studies differed in the histological scoring system for staging fibrosis in NAFLD patients. The majority of studies utilized the NASH CRN scoring system, while one study relied on METAVIR criteria [26]. The scores from this study were converted to their NASH CRN equivalent for the meta-analysis [53] (see Supplementary Table S6 for correspondence between the NASH CRN and the METAVIR systems).

Not all studies provided detailed information about the biopsy. All except one [49] reported the length of the biopsy samples, which ranged from 13.8 to 27 mm. None of the studies reported the size of the needle gauge. Biopsy samples had been evaluated by a single pathologist in five studies [25,46,48,51,52], and one study reported evaluations by a pathology consortium and centralized pathologists [26]. One other study did not report information regarding the histology assessment [49]. Only one study had relied on hepatopathologists for their histopathology assessments [48]. More details about the biopsy characteristics of each study are available in Supplementary Table S7.

#### 3.1.3. Methodological Quality Assessment

The methodological quality assessment results are summarized in Supplementary Figures S1 and S2. Only one study was judged to be at low risk of bias in all four domains of the QUADAS-2 instrument [48]. Four studies were scored at a high risk of bias for the patient selection domain [25,26,46,49]. In three of them, healthy controls had been included [25,46,49]. The study reports did not specify whether the participants had been enrolled as a consecutive series, based on random sampling, or as a convenience sample [25,46,49]. In one other study, the analysis was performed on data collected in three validation studies, performed independently, with considerable heterogeneity in the respective study populations [26].

Three studies had included only diabetic patients or morbidly obese patients, qualifying for bariatric surgery. Due to these inclusion criteria, the respective study groups are not representative of the general population of suspected NAFLD patients. We therefore had applicability concerns in the patient selection domain for a number of studies [25,26,46,49,52].

Three studies did not report whether the threshold value for FibroTest™ was pre-specified before data analysis. We scored the index test-related risk of bias for these studies as “unclear” [25,51,52]. One study failed to report whether the data evaluation process was blinded, and that study also did not report the time interval between the sample collection and biopsy [49]. We scored the risk of bias of this study as “unclear” for both the reference standard and the flow and timing domain.

### 3.2. Performance of FibroTest™

#### 3.2.1. Accuracy of FibroTest™ for Detecting Advanced Fibrosis (≥F3)

In total, five studies were included in the meta-analysis of the diagnostic accuracy of FibroTest™ in detecting advanced fibrosis. These studies had recruited 2103 NAFLD participants in total, of which 551 had advanced fibrosis. Three of the studies reported the performance of the test at a single threshold, and the other two studies reported accuracy data at multiple different thresholds (see Supplementary Figure S3 for forest plots).

**Figure 2:**
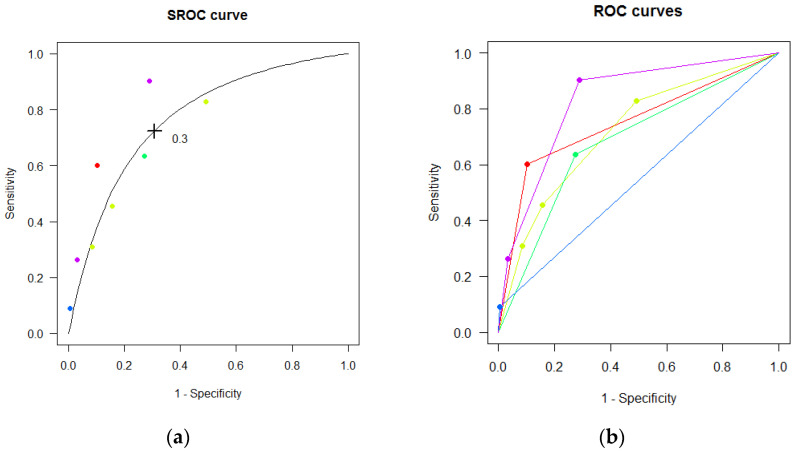
Multiple thresholds (**a**) SROC and (**b**) ROC curves for FibroTest in detecting advanced fibrosis. Each point represents a reported threshold value, points of the same color represent thresholds reported within the same study. The cross in the SROC curve indicates the Youden-based threshold value: Youden-threshold: 0.30, sensitivity: 0.72 (95% CI: 0.52; 0.87), specificity: 0.69 (95% CI: 0.49; 0.84), AUC: 0.77 (95% CI: 0.64; 0.86).

With the multiple thresholds model, our summary estimate of AUC was 0.77 (95% CI: 0.64 to 0.86), with a mean sensitivity of 0.72 (95% CI: 0.52 to 0.87) and mean specificity of 0.69 (95% CI: 0.49 to 0.84) at the Youden-index maximizing threshold of 0.30 (Figure 2).

We used the multiple thresholds model to calculate expected positive and negative predictive values for desired levels of sensitivity and specificity, examining advanced fibrosis prevalences between 10% and 50%. The results are shown in Table 2.

**Figure 3:**
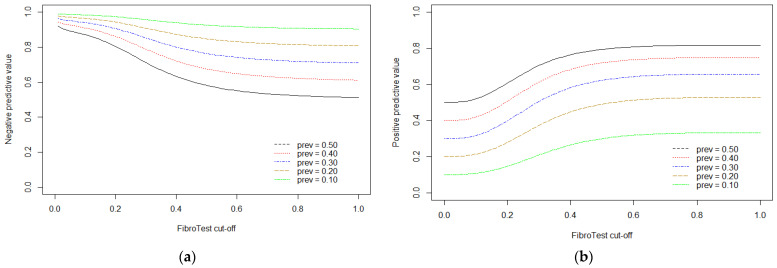
Plots illustrating the negative (**a**) and positive (**b**) predictive values of FibroTest for detecting advanced fibrosis at corresponding threshold values, projected by the multiple thresholds model. Each colored line represents a different prevalence setting, ranging from 10% to 50%.

By fixing sensitivity at high values (from 0.90 to 0.98), we observed corresponding specificity values that ranged from 0.42 to 0.13, at threshold values of 0.20 to 0.12. As expected, the projected positive predictive values (PPVs) were higher in settings with a higher disease prevalence, however, the estimated negative predictive values (NPVs) were acceptable (≥0.80) at all prevalences. On the other hand, setting specificity at values between 0.90 and 0.98 resulted in sensitivities between 0.37 and 0.09, at threshold values of 0.48 to 0.86, and acceptable PPVs (≥0.80) only in settings with disease prevalence of 50%. Figure 3 illustrates the corresponding PPV and NPV for different thresholds based on the multiple thresholds model using all available information for advanced fibrosis.

#### 3.2.2. Accuracy of FibroTest™ for Detecting Significant Fibrosis (≥F2)

**Figure 4:**
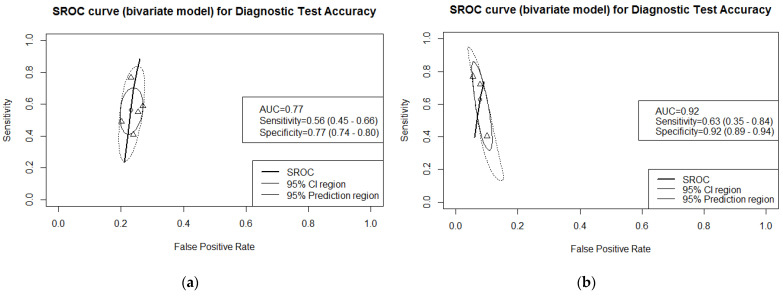

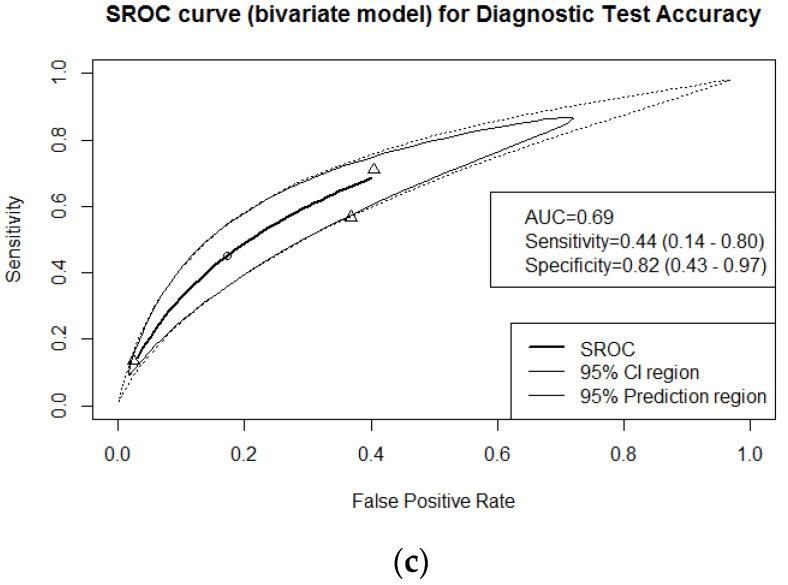
SROC curves for FibroTest in detecting significant fibrosis (**a**), cirrhosis (**b**), and any fibrosis level (**c**). In each plot, the circle (summary point) represents the summary estimate of sensitivity and specificity. Each triangle represents a single threshold value reported from an included study. The solid eclipse represents the 95% confidence region, and the dotted eclipse represents the prediction region. AUC: area under the receiver operating curve, 50%.

Five studies, reporting on 1788 NAFLD participants, of which 952 patients had significant fibrosis, reported the performance of FibroTest™ in detecting significant fibrosis. (see Supplementary Figure S4 for the forest plots at reported thresholds, ranging from 0.30 to 0.75). The resulting AUC was 0.77 in our meta-analysis. See Figure 4a for SROC curve and corresponding 95% CI and prediction region.

#### 3.2.3. Accuracy of FibroTest™ in Detecting Cirrhosis (F4 vs. F0–F3)

Three studies (1370 participants, 177 with cirrhosis) reported on the accuracy of FibroTest™ in detecting cirrhosis. The proportion of study participants with cirrhosis ranged from 10% to 13%. The studies reported accuracy for different cut-offs: 0.57, 074, and 0.75. The estimate of the AUC in the meta-analysis was 0.92. See Supplementary Figure S5 for forest plots of these studies and Figure 4b for the ROC curve.

#### 3.2.4. Accuracy of FibroTest™ in Detecting any Fibrosis (F1–4 vs. F0)

Three studies reported the performance of FibroTest™ for detecting any level of fibrosis in NAFLD patients. These studies had recruited 1583 participants, of which 1214 had some level of fibrosis. The cut-offs for accuracy estimates were not the same: 0.27 was used in two studies and 0.26 in one. See Supplementary Figure S6 for the forest plots. Our summary estimate of the AUC was 0.69. (see Figure 4c).

### 3.3. Sensitivity Analysis

We conducted sensitivity analyses to examine the impact of the different scoring systems for staging liver fibrosis on the meta-analytic findings. Removing the one study with METAVIR criteria from the meta-analysis for advanced fibrosis did not meaningfully affect the results (AUC: 0.77, sensitivity: 0.73, and specificity: 0.69).

We also did not observe any substantial differences when comparing the results of the meta-analysis for significant fibrosis with and without including this study. Including this study resulted in a slightly lower AUC (0.71 vs. 0.77) and small changes in the summary estimates of sensitivity (0.49 vs. 0.56) and specificity (0.82 vs. 0.77)

## 4. Discussion

### 4.1. Summary of Main Findings

In this systematic review, summarizing the evidence from seven studies, FibroTest™ did not meet the minimally acceptable performance level in detecting significant (≥F2), advanced (≥F3), or any fibrosis (AUC: 0.77, 0.77, and 0.69, respectively). In comparison, the diagnostic accuracy of the test in detecting cirrhosis (F4) was more promising, demonstrating an AUC of 0.92.

In meta-analysis of advanced fibrosis, where the studies reported different thresholds, we could use the multiple thresholds model to calculate negative and positive predictive values for a range of prevalences of the disease, optimizing predefined sensitivity and specificity values. This analysis showed that by optimizing sensitivity to values above 0.90, the test could result in high NPVs (>90%) in settings with low prevalence of disease, such as primary and secondary care settings, but with relatively low PPVs (11–61%).

### 4.2. Strengths and Limitations of the Review

FibroTest™ is a continuous linear biochemical assessment of NAFLD progression, providing a quantitative estimate of liver fibrosis that is usually interpreted relative to the METAVIR scoring system (F0 to F4) [29] (Supplementary Table S8) [28]. In our meta-analysis, only one of the seven included studies had used METAVIR, and the others used the NASH CRN scoring system. To incorporate all available data, we converted the METAVIR scores into the corresponding NASH CRN equivalent (Supplementary Table S6). After the conversion, one primary study moved from the significant fibrosis group to the advanced fibrosis group, changing the target condition from what was originally reported in the paper.

It should be noted that our meta-analysis results are based on test accuracy data reported by primary studies conducted in settings with a disease prevalence that exceeds that in most primary care settings. The limited number of studies available for meta-analysis impeded all subgroup analyses or formal explorations of sources of heterogeneity, including those related to prevalence, age, sex, and comorbidity. Further studies with sufficient individual patient data are required for conducting subgroup analysis and drawing valid conclusions about differences in performance across identifiable subgroups of NAFLD patients. Moreover, all studies included in our meta-analysis were performed in western countries. This may limit the generalizability of our findings and indicates a need for further evaluations of test performance in different ethnicities.

Although a list of recommended cut-offs was published for detecting different levels of fibrosis (Supplementary Table S8), studies were not consistent in using the same thresholds for each target condition, which is another factor related to variability in reported performance measures. We used the novel multiple thresholds meta-analysis model, which enabled us to use all available data and reduce the risk of an optimistic evaluation of the biomarker.

Information about the histological procedure, such as size of the needle gauge, the length of the biopsy, and number of portal tracts, were often not reported. However, these factors affect the reliability of the reference standard.

### 4.3. Other Published Systematic Reviews

Since FibroTest™ was originally developed for the assessment of fibrosis in HCV patients, most of the available accuracy studies were performed in patients with viral hepatitis. A limited number of studies evaluated the performance of the test in NAFLD patients. A recently published systematic review, with a focus on the obese population (BMI over 30), assessed the performance of a number of non-invasive tests, including FibroTest™. It reported higher accuracy estimates than those obtained in our review. In their systematic review, they separately pooled and reported results of studies that used low and high thresholds of FibroTest™. Their meta-analysis of two studies reporting the performance of the test in detecting significant fibrosis using low thresholds, resulted in pooled sensitivity and specificity of 0.67 (95% CI: 0.59 to 0.74) and 0.75 (95% CI: 0.70 to 0.80), respectively. While for the same target condition but in the high thresholds, the meta-analysis resulted in sensitivity and specificity of 0.13 (95% CI: 0.07 to 0.22) and 0.99 (95% CI: 0.98 to 0.99), respectively. For advanced fibrosis, the study reported slightly higher sensitivity for both meta-analysis of low and high thresholds, 0.83 (95% CI: 0.77 to 0.88) and 0.46 (95% CI: 0.35 to 0.57), and lower specificity, 0.63 (95% CI: 95% CI: 0.59 to 0.67) and 0.94 (95% CI: 0.92 to 0.96), respectively. [6] By comparing the test with other single biomarkers, the authors suggested that the complex panels, including FibroTest™, can perform more accurately. Unfortunately, this review had included only a small number of studies and did not consider the variability in histological scoring systems in the included primary studies.

Two other systematic reviews have reported slightly higher accuracy levels for FibroTest™ in detecting significant fibrosis: an AUC of 0.78 (95% CI: 0.72 to 0.85) and 0.84 (95% CI: 0.76 to 0.92) [54,55]. These reviews were based on the results of two and one primary study only, respectively.

### 4.4. Implications

FibroTest™ is a panel of markers recommended by the WHO [56], the American Association for the Study of Liver Diseases (AASLD) [57,58], the European Association for the Study of the Liver (EASL) [12], and the Asia-Pacific Association for the Study of the Liver (APASL) [59] for evaluating hepatic fibrosis in patients with viral hepatitis. It was also shown to have high predictive values for significant lesions in ALD patients [24]. Due to the similarity of fibrosis features between ALD and NAFLD patients, it was then proposed for evaluating fibrosis levels in NAFLD patients [25].

FibroTest™ is currently available in many countries and usually used in combination with other blood tests, including SteatoTest for steatosis grading and ActiTest for inflammation activity grading. The EASL clinical practice guideline (2016) recommends that surrogate non-invasive markers of fibrosis, such as NAFLD Fibrosis Score (NFS), Enhanced Liver Fibrosis (ELF), and FibroTest™, which have acceptable diagnostic accuracy (AUC > 0.80), should be used in NAFLD patients, to rule out significant fibrosis [16]. This recommendation was based on only two studies that evaluated FibroTest™. In our systematic review, based on five studies, including more recent ones, we observed a lower AUC (0.77). Our meta-analysis results showed that FibroTest™ has acceptable diagnostic performance only in detecting cirrhosis in NAFLD patients (AUC: 0.92).

Other recommended non-invasive markers in NAFLD patients have also been documented in the literature, with variable performance levels in detecting degrees of fibrosis. For instance, ELF showed acceptable accuracy (≥0.80) in detecting significant and advanced fibrosis [60]. However, like FibroTest™, better diagnostic performance of the test at higher thresholds and in high-prevalence settings suggests careful consideration of the likely disease prevalence in the intended use setting and adoption of suitable test thresholds to achieve the desired test performance.

In addition to serum-based markers, other tests, such as those based on elastography, have been described in the literature, often with promising diagnostic performance [6]. A recently published comparative study evaluated the most validated fibrosis tests, including Fibrosis-4 (FIB-4), NFS, FibroTest™, and Fibroscan. The findings of the study showed significantly better performance of Fibroscan in detecting NAFLD-related advanced fibrosis compared to all other evaluated blood tests [48]. This indicates that, when available, elastography-based tests can be useful as first-line procedures as they give an immediate result after a quick and easy-to-perform examination [48]. Yet, difficulties in performing these tests in obese patients, limitations in distinguishing between steatosis and steatohepatitis, and a lack of sufficient paired studies make comparisons with other markers difficult.

A few accuracy studies have evaluated the performance of FibroTest™ in comparison to other blood tests in detecting different stages of fibrosis [46,51,52]. One study showed that more complex models, such as Hepascore, FibroTest™, and FIB-4, can identify advanced fibrosis in NAFLD patients significantly better than simple models, such as the Platelet Ratio Index (APRI) [51]. Yet another comparison showed that none of the commonly used approaches, including FibroTest™, FIB4, APRI, and NFS, performed significantly better than plasma aspartate aminotransferase (AST) in detecting diabetic NAFLD patients with advanced fibrosis [46]. These inconsistent conclusions highlight the need for further well-designed comparisons in the intended use population. More comparative accuracy studies of high methodological quality are necessary for a valid appraisal of the performance of FibroTest™ relative to other non-invasive markers.

## 5. Conclusions

Our meta-analysis of the available evidence showed acceptable diagnostic performance (AUC > 0.80) of FibroTest™ only in detecting cirrhosis. EASL-EASD-EASO Clinical Practice Guidelines recommended FibroTest™ as a non-invasive test with acceptable diagnostic accuracy for detecting fibrosis and NAFLD progression [16]. In primary, secondary, and tertiary settings, with a 10–50% disease prevalence, FibroTest™ can have a high NPV, based on sensitives between 0.90 and 0.98, demonstrating its ability to rule out advanced fibrosis in NAFLD patients. However, clinicians should notice the low specificity at the corresponding thresholds, leading to a considerable number of false positive results, potentially resulting in invasive and expensive follow-up evaluations, such as liver biopsy. Since these were projections, further studies are needed, conducted in primary care settings.

## Figures and Tables

**Table 1:** Major characteristics of the 10 studies included.

Study ID	Setting	Population	N (Number of Females)	Age, y	BMI	Target Condition/N(%)	Scoring System	Liver Enzymes		DM
								AST	ALT	
Bril 2020 [46]	Outpatients Hospital	Suspected NAFLD	162 (28)	57	34.5	Advanced fibrosis (F ≥ 3)/31(19%)	NASH CRN	50.9	54.7	100%
Lardi 2020 * [47]	Outpatients Hospital	Biopsy-proven NAFLD	73 (NR)	NR	NR	Advanced fibrosis (F ≥ 3)/NR	NR	NR	NR	NR
Boursier 2019 [48]	Outpatients Hospital	Biopsy-proven NAFLD	938 (408)	56.5	31.8	Significant fibrosis (F ≥ 2)/635(68%) Advanced fibrosis (F ≥ 3)/383(41%) Cirrhosis (F4)/126(13%) Any fibrosis (F ≥ 1)/849(91%)	NASH CRN	39 ^α^	56 ^α^	58.5%
Bril 2019 ^¥^ [49]	Outpatients Hospital	Suspected NAFLD	151 (64)	57	34.7	Significant fibrosis (F ≥ 2)/50(33%) Any fibrosis (F ≥ 1)/113(75%)	NASH CRN	41	57	100%
Stael 2019 * [50]	NR	Suspected NAFLD	268 (NR)	NR	NR	NAS ≥ 4 and Significant fibrosis (F2–4)/NR	NR	NR	NR	100%
Munteanu2016 * [28]	Outpatients Hospital	Suspected NAFLD	600 (220)	53.2 ^α^	NR	Any fibrosis (F ≥ 1)/286(48%)	NASH CRN	NR	NR	22.7%
Poynard 2012 [26]	Inpatient, Hospital	Suspected NAFLD	494 (383)	42.2	47.4	Advanced fibrosis (F ≥ 3)/49(10%) Any fibrosis (F ≥ 1)/252(51%)	METAVIR	NR	36	28.5%
Adams 2011 [51]	Outpatients Hospital	Biopsy-proven NAFLD	242 (96)	46.8	30.2	Significant fibrosis (F ≥ 2)/97(40%) Advanced fibrosis (F ≥ 3)/53(22%) Cirrhosis (F4)/25(10%)	NASH CRN	37	66.5	24.8%
Sebastiani 2011 [52]	Outpatients Hospital	Biopsy-proven NASH	190 (48)	51.2	28.9	Significant fibrosis (F ≥ 2)/98(52%) Cirrhosis (F4)/25(14%)	NASH CRN	79.6	72.3	NR
Ratziu 2006 [25]	Inpatients Hospital	Suspected NAFLD	267 (112)	51.2	40% > 27	Significant fibrosis (F ≥ 2)/73(27%) Advanced fibrosis (F ≥ 3)/35(13%)	NASH CRN	48.2	73.9	34%

N: total number of patients, NR: not reported, DM: diabetes mellitus, ALT: alanine aminotransferase, ALT: aspartate aminotransferase. ^α^ Median. * Three studies were excluded from the meta-analysis due to lack of information on cut-offs and/or data for reconstructing 2-by-2 tables. ^¥^ Data on advanced fibrosis was also reported in this paper; however, the most recent data reported by the authors in the same population was selected for the meta-analysis.

**Table 2:** Projected predictive values for different prevalences of advanced fibrosis at selected levels of sensitivity and selected levels of specificity.

**Prevalence**	**Fixed 0.90 Sensitivity**				**Fixed 0.95 Sensitivity**				**Fixed 0.98 Sensitivity**			
	**Cut-Off**	**Specificity**	**PPV**	**NPV**	**Cut-Off**	**Specificity**	**PPV**	**NPV**	**Cut-Off**	**Specificity**	**PPV**	**NPV**
**0.10**	0.20	0.42	0.15	0.97	0.16	0.26	0.13	0.98	0.12	0.13	0.11	0.98
**0.20**			0.28	0.94			0.24	0.95			0.22	0.96
**0.30**			0.40	0.91			0.36	0.92			0.32	0.94
**0.40**			0.51	0.86			0.46	0.89			0.43	0.90
**0.50**			0.61	0.81			0.56	0.84			0.53	0.86
	**Fixed 0.90 Specificity**				**Fixed 0.95 Specificity**				**Fixed 0.98 Specificity**			
	**Cut-Off**	**Specificity**	**PPV**	**NPV**	**Cut-Off**	**Specificity**	**PPV**	**NPV**	**Cut-Off**	**Specificity**	**PPV**	**NPV**
**0.10**	0.48	0.37	0.29	0.93	0.62	0.21	0.32	0.92	0.86	0.09	0.33	0.91
**0.20**			0.48	0.85			0.52	0.83			0.53	0.81
**0.30**			0.62	0.77			0.65	0.74			0.66	0.72
**0.40**			0.71	0.68			0.74	0.64			0.75	0.62
**0.50**			0.79	0.59			0.81	0.55			0.82	0.52

PPV: positive predictive value, NPV: negative predictive value.

## Data Availability

The datasets used and analyzed during the current study are available from the corresponding author on reasonable request.

## Supplementary Materials

The following are available online at https://www.mdpi.com/article/10.3390/jcm10112415/s1, Supplementary Table S1: PRISMA-DTA checklist. Supplementary Table S2: Preliminary search strategy for Medline. Supplementary Table S3: Conversion grid for the stages of liver fibrosis. Supplementary Table S4: Histological scoring systems developed to characterize changes in NAFLD progression. Supplementary Table S5: Number of excluded papers for each exclusion reason. Supplementary Table S6: Correspondence between the NASH CRN and the METAVIR systems, reported by Boursier et al. 2017. Supplementary Table S7: Biopsy criteria of studies included in the meta-analysis. Supplementary Table S8: METAVIR scoring systems and pre-determined FibroTest cut-offs. Supplementary Figure S1: Graphical summary of the methodological quality of included studies using the QUADAS-2 tool. Supplementary Figure S2: Methodological quality of each of the included studies per domain of the QUADAS-2 tool.. Supplementary Figure S3: Forest plot of all included studies for advanced fibrosis. FN = false negative; FP = false positive; TN = true negative; TP = true positive. The forest plot shows an estimate of sensitivity and specificity from each study and the threshold used. The horizontal lines around each box depict the confidence intervals. Studies with more than one threshold are labeled with letters. Supplementary Figure S4: Forest plot of studies investigating diagnostic accuracy of FibroTest for detecting significant fibrosis. FN = false negative; FP = false positive; TN = true negative; TP = true positive. The forest plot shows an estimate of sensitivity and specificity from each study and the threshold used. The horizontal lines around each box depict the confidence intervals. Supplementary Figure S5: Forest plot of studies investigating diagnostic accuracy of FibroTest for detecting cirrhosis. FN = false negative; FP = false positive; TN = true negative; TP = true positive. The forest plot shows an estimate of sensitivity and specificity from each study and the threshold used. The horizontal lines around each box depict the confidence intervals. Supplementary Figure S6: Forest plots of studies investigating diagnostic accuracy of FibroTest for detecting any fibrosis. FN = false negative; FP = false positive; TN = true negative; TP = true positive. The forest plot shows an estimate of sensitivity and specificity from each study and the threshold used. The horizontal lines around each box depict the confidence intervals.

## References

[1] Younossi Z.M., Koenig A.B., Abdelatif D., Fazel Y., Henry L., Wymer M. (2016). Global epidemiology of nonalcoholic fatty liver disease-Meta-analytic assessment of prevalence, incidence, and outcomes. Hepatology.

[2] Younossi Z.M., Stepanova M., Afendy M., Fang Y., Younossi Y., Mir H., Srishord M. (2011). Changes in the Prevalence of the Most Common Causes of Chronic Liver Diseases in the United States From 1988 to 2008. Clin. Gastroenterol. Hepatol..

[3] Adams L.A., Anstee Q.M., Tilg H., Targher G. (2017). Non-alcoholic fatty liver disease and its relationship with cardiovascular disease and other extrahepatic diseases. Gut.

[4] Hadi A., Mohammadi H., Miraghajani M., Ghaedi E. (2018). Efficacy of synbiotic supplementation in patients with nonalcoholic fatty liver disease: A systematic review and meta-analysis of clinical trials: Synbiotic supplementation and NAFLD. Crit. Rev. Food Sci. Nutr..

[5] Fazel Y., Koenig A.B., Sayiner M., Goodman Z.D., Younossi Z.M. (2016). Epidemiology and natural history of non-alcoholic fatty liver disease. Metabolism.

[6] Ooi G.J., Mgaieth S., Eslick G.D., Burton P.R., Kemp W.W., Roberts S.K., Brown W.A. (2018). Systematic review and meta-analysis: Non-invasive detection of non-alcoholic fatty liver disease related fibrosis in the obese. Obes. Rev..

[7] Singh S., Allen A.M., Wang Z., Prokop L.J., Murad M.H., Loomba R. (2015). Fibrosis Progression in Nonalcoholic Fatty Liver vs Nonalcoholic Steatohepatitis: A Systematic Review and Meta-analysis of Paired-Biopsy Studies. Clin. Gastroenterol. Hepatol..

[8] Dulai P.S., Singh S., Patel J., Soni M., Prokop L.J., Younossi Z., Loomba R. (2017). Increased risk of mortality by fibrosis stage in nonalcoholic fatty liver disease: Systematic review and meta-analysis. Hepatology.

[9] Angulo P., Kleiner D.E., Dam-Larsen S., Adams L.A., Björnsson E.S., Charatcharoenwitthaya P., Mills P.R., Keach J.C., Lafferty H.D., Stahler A. (2015). Liver Fibrosis, but No Other Histologic Features, Is Associated with Long-term Outcomes of Patients With Nonalcoholic Fatty Liver Disease. Gastroenterology.

[10] Ekstedt M., Hagström H., Nasr P., Fredrikson M., Stål P., Kechagias S., Hultcrantz R. (2015). Fibrosis stage is the strongest predictor for disease-specific mortality in NAFLD after up to 33 years of follow-up. Hepatology.

[11] Taylor R.S., Taylor R.J., Bayliss S., Hagstrom H., Nasr P., Schattenberg J.M., Ishigami M., Toyoda H., Wong V., Peleg N. (2020). Association Between Fibrosis Stage and Outcomes of Patients with Non-Alcoholic Fatty Liver Disease: A Systematic Review and Meta-Analysis. Gastroenterology.

[12] European Association for Study of Liver, Asociacion Latinoamericana para el Estudio del Higado (2015). EASL-ALEH Clinical Practice Guidelines: Non-invasive tests for evaluation of liver disease severity and prognosis. J. Hepatol..

[13] European Association for the Study of the Liver (EASL), European Association for the Study of Diabetes (EASD), European Association for the Study of Obesity (EASO) (2016). EASL-EASD-EASO Clinical Practice Guidelines for the management of non-alcoholic fatty liver disease. Obes. Facts.

[14] Chalasani N., Younossi Z., LaVine J.E., Charlton M., Cusi K., Rinella M., Harrison S.A., Brunt E.M., Sanyal A.J. (2018). The diagnosis and management of nonalcoholic fatty liver disease: Practice guidance from the American Association for the Study of Liver Diseases. Hepatology.

[15] Rinella M.E., Tacke F., Sanyal A.J., Anstee Q.M. (2019). Report on the AASLD/EASL joint workshop on clinical trial endpoints in NAFLD. J Hepatol..

[16] European Association for the Study of the Liver (EASL), European Association for the Study of Diabetes (EASD), European Association for the Study of Obesity (EASO) (2016). EASL-EASD-EASO Clinical Practice Guidelines for the management of non-alcoholic fatty liver disease. J. Hepatol..

[17] Bravo A.A., Sheth S.G., Chopra S. (2001). Liver Biopsy. N. Engl. J. Med..

[18] The Royal College of Pathologists (2008). Tissue Pathways for Liver Biopsies for the Investigation of Medical Disease and for Focal Lesions.

[19] Ratziu V., Charlotte F., Heurtier A., Gombert S., Giral P., Bruckert E., Grimaldi A., Capron F., Poynard T. (2005). Sampling Variability of Liver Biopsy in Nonalcoholic Fatty Liver Disease. Gastroenterology.

[20] Poynard T., Imbert-Bismut F., Munteanu M., Messous D., Myers R.P., Thabut D., Ratziu V., Mercadier A., Benhamou Y., Hainque B. (2004). Overview of the diagnostic value of biochemical markers of liver fibrosis (FibroTest, HCV FibroSure) and necrosis (ActiTest) in patients with chronic hepatitis C. Comp. Hepatol..

[21] Myers R.P., Tainturier M.-H., Ratziu V., Piton A., Thibault V., Imbert-Bismut F., Messous D., Charlotte F., Di Martino V., Benhamou Y. (2003). Prediction of liver histological lesions with biochemical markers in patients with chronic hepatitis B. J. Hepatol..

[22] Poynard T., Zoulim F., Ratziu V., Degos F., Imbert-Bismut F., Deny P., Landais P., El Hasnaoui A., Slama A., Blin P. (2005). Longitudinal Assessment of Histology Surrogate Markers (FibroTest-ActiTest) During Lamivudine Therapy in Patients with Chronic Hepatitis B Infection. Am. J. Gastroenterol..

[23] Calès P., Oberti F., Michalak S., Hubert-Fouchard I., Rousselet M.C., Konaté A., Gallois Y., Ternisien C., Chevailler A., Lunel F. (2005). A novel panel of blood markers to assess the degree of liver fibrosis. Hepatology.

[24] Naveau S., Raynard B., Ratziu V., Abella A., Imbert-Bismuth F., Messous D., Beuzen F., Capron F., Thabut D., Munteanu M. (2005). Biomarkers for the prediction of liver fibrosis in patients with chronic alcoholic liver disease. Clin. Gastroenterol. Hepatol..

[25] Ratziu V., Massard J., Charlotte F., Messous D., Imbert-Bismut F., Bonyhay L., Tahiri M., Munteanu M., Thabut D., Cadranel J.F. (2006). Diagnostic value of biochemical markers (FibroTest-FibroSURE) for the prediction of liver fibrosis in patients with non-alcoholic fatty liver disease. BMC Gastroenterol..

[26] Poynard T., Lassailly G., Diaz E., Clement K., Caïazzo R., Tordjman J., Munteanu M., Perazzo H., Demol B., Callafe R. (2012). Performance of Biomarkers FibroTest, ActiTest, SteatoTest, and NashTest in Patients with Severe Obesity: Meta Analysis of Individual Patient Data. PLoS ONE.

[27] Moher D., Liberati A., Tetzlaff J., Altman D.G. (2009). Preferred Reporting Items for Systematic Reviews and Meta-Analyses: The PRISMA Statement. Ann. Intern. Med..

[28] Munteanu M., Tiniakos D., Anstee Q., Charlotte F., Marchesini G., Bugianesi E., Trauner M., Gomez M.R., Oliveira C., Day C. (2016). Diagnostic performance of FibroTest, SteatoTest and ActiTest in patients with NAFLD using the SAF score as histological reference. Aliment. Pharmacol. Ther..

[29] Poynard T., Imbert-Bismut F., Munteanu M., Ratziu V. (2005). FibroTest-FibroSURE™: Towards a universal biomarker of liver fibrosis?. Expert Rev. Mol. Diagn..

[30] Kleiner D.E., Brunt E.M., Van Natta M., Behling C., Contos M.J., Cummings O.W., Ferrell L.D., Liu Y.-C., Torbenson M.S., Unalp-Arida A. (2005). Design and validation of a histological scoring system for nonalcoholic fatty liver disease. Hepatology.

[31] The French METAVIR Cooperative Study Group (1994). Intraobserver and interobserver variations in liver biopsy interpretation in patients with chronic hepatitis C. Hepatology.

[32] Batts K.P., Ludwig J. (1995). Chronic hepatitis. An update on terminology and reporting. Am. J. Surg. Pathol..

[33] Bedossa P., Arola J., Susan D., Gouw A., Maria G., Lackner K., Schirmacher P., Terracciano L., Anstee Q., Ratziu V. (2018). The EPoS staging system is a reproducible 7-tierfibrosis score for NAFLD adapted both to glass slides and digitized images (e-slides). J. Hepatol..

[34] Desmet V.J., Gerber M., Hoofnagle J.H., Manns M., Scheuer P.J. (1994). Classification of chronic hepatitis: Diagnosis, grading and staging. Hepatology.

[35] Ishak K., Baptista A., Bianchi L., Callea F., De Groote J., Gudat F., Denk H., Desmet V., Korb G., Macsween R.N. (1995). Histological grading and staging of chronic hepatitis. J. Hepatol..

[36] Knodell R.G., Ishak K.G., Black W.C., Chen T.S., Craig R., Kaplowitz N., Kiernan T.W., Wollman J. (1981). Formulation and application of a numerical scoring system for assessing histological activity in asymptomatic chronic active hepatitis. Hepatology.

[37] Scheuer P.J. (1991). Classification of chronic viral hepatitis: A need for reassessment. J. Hepatol..

[38] Bedossa P., Poitou C., Veyrie N., Bouillot J.-L., Basdevant A., Paradis V., Tordjman J., Clement K. (2012). Histopathological algorithm and scoring system for evaluation of liver lesions in morbidly obese patients. Hepatology.

[39] Brunt E.M., Janney C.G., Di Bisceglie A.M., Neuschwander-Tetri B.A., Bacon B.R. (1999). Nonalcoholic steatohepatitis: A proposal for grading and staging the histological lesions. Am. J. Gastroenterol..

[40] Matteoni C.A., Younossi Z.M., Gramlich T., Boparai N., Liu Y.C., McCullough A.J. (1999). Nonalcoholic fatty liver disease: A spectrum of clinical and pathological severity. Gastroenterology.

[41] Younossi Z.M., Stepanova M., Rafiq N., Makhlouf H., Younoszai Z., Agrawal R., Goodman Z. (2011). Pathologic criteria for nonalcoholic steatohepatitis: Interprotocol agreement and ability to predict liver-related mortality. Hepatology.

[42] Whiting P.F., Rutjes A.W., Westwood M.E., Mallett S., Deeks J.J., Reitsma J.B., Leeflang M.M., Sterne J.A., Bossuyt P.M.M., The QUADAS-2 Group (2011). QUADAS-2: A Revised Tool for the Quality Assessment of Diagnostic Accuracy Studies. Ann. Intern. Med..

[43] Bürkner P.C., Doebler P. (2014). Testing for publication bias in diagnostic meta-analysis: A simulation study. Stat. Med..

[44] Schneider A., Linde K., Reitsma J.B., Steinhauser S., Rucker G. (2017). A novel statistical model for analyzing data of a systematic review generates optimal cutoff values for fractional exhaled nitric oxide for asthma diagnosis. J. Clin. Epidemiol..

[45] Steinhauser S., Schumacher M., Rücker G. (2016). Modelling multiple thresholds in meta-analysis of diagnostic test accuracy studies. BMC Med. Res. Methodol..

[46] Bril F., McPhaul M.J., Caulfield M.P., Clark V.C., Soldevilla-Pico C., Firpi-Morell R.J., Lai J., Shiffman D., Rowland C.M., Cusi K. (2020). Performance of Plasma Biomarkers and Diagnostic Panels for Nonalcoholic Steatohepatitis and Advanced Fibrosis in Patients with Type 2 Diabetes. Diabetes Care.

[47] Lardi L.L., Lul R.M., Port G.Z., Coral G.P., Peres A., Dorneles G.P., Branco F., Fernandes S., Leães C.G., Mattos A.A. (2020). Fibromax and inflamatory markers cannot replace liver biopsy in the evaluation of non-alcoholic fatty liver disease. Minerva Gastroenterol. Dietol..

[48] Boursier J., Guillaume M., Leroy V., Irlès M., Roux M., Lannes A., Foucher J., Zuberbuhler F., Delabaudière C., Barthelon J. (2019). New sequential combinations of non-invasive fibrosis tests provide an accurate diagnosis of advanced fibrosis in NAFLD. J. Hepatol..

[49] Bril F., McPhaul M.J., Caulfield M.P., Castille J.-M., Poynard T., Soldevila-Pico C., Clark V.C., Firpi-Morell R.J., Lai J., Cusi K. (2019). Performance of the SteatoTest, ActiTest, NashTest and FibroTest in a multiethnic cohort of patients with type 2 diabetes mellitus. J. Investig. Med..

[50] Staels B., Ratziu V., Francque S., Harrison S.A., Bedossa P., Roudot A., Majd Z., Brozek J., Bem-Sudirk F., Birman P. (2019). 279-LB: NIS4, a Novel Blood Test, Can Identify" At-Risk" NASH (NAS> 4 and Fibrosis> 2) in T2D Patients. Am. Diabetes Assoc..

[51] Adams L.A., George J., Bugianesi E., Rossi E., De Boer W.B., Van Der Poorten D., Ching H.L., Bulsara M., Jeffrey G.P. (2011). Complex non-invasive fibrosis models are more accurate than simple models in non-alcoholic fatty liver disease. J. Gastroenterol. Hepatol..

[52] Sebastiani G., Castera L., Halfon P., Pol S., Mangia A., Di Marco V., Pirisi M., Voiculescu M., Bourliere M., Alberti A. (2011). The impact of liver disease aetiology and the stages of hepatic fibrosis on the performance of non-invasive fibrosis biomarkers: An international study of 2411 cases. Aliment. Pharmacol. Ther..

[53] Boursier J., de Ledinghen V., Leroy V., Anty R., Francque S., Salmon D., Lannes A., Bertrais S., Oberti F., Fouchard-Hubert I. (2017). A stepwise algorithm using an at-a-glance first-line test for the non-invasive diagnosis of advanced liver fibrosis and cirrhosis. J. Hepatol..

[54] Musso G., Gambino R., Cassader M., Pagano G. (2010). Meta-analysis: Natural history of non-alcoholic fatty liver disease (NAFLD) and diagnostic accuracy of non-invasive tests for liver disease severity. Ann. Med..

[55] Poynard T., Morra R., Halfon P., Castera L., Ratziu V., Imbert-Bismut F., Naveau S., Thabut D., Lebrec D., Zoulim F. (2007). Meta-analyses of FibroTest diagnostic value in chronic liver disease. BMC Gastroenterol..

[56] World Health Organization (2014). Guidelines for the Screening, Care and Treatment of Persons with Hepatitis C Infection.

[57] Panel A.I., Chung R.T., Davis G.L., Jensen D.M., Masur H., Saag M.S. (2015). Hepatitis C guidance: AASLD-IDSA recommendations for testing, managing, and treating adults infected with hepatitis C virus. Hepatology.

[58] Terrault N.A., Lok A.S., McMahon B.J., Chang K.-M., Hwang J., Jonas M.M., Bzowej N.H., Wong J.B. (2018). Update on prevention, diagnosis, and treatment of chronic hepatitis B: AASLD 2018 hepatitis B guidance. Hepatology.

[59] Shiha G., Sarin S.K., Ibrahim A.E., Omata M., Kumar A., Lesmana L.A., Leung N., Tozun N., Hamid S., Jafri W. (2009). Liver fibrosis: Consensus recommendations of the Asian Pacific Association for the Study of the Liver (APASL). Hepatol. Int..

[60] Vali Y., Lee J., Boursier J., Spijker R., Löffler J., Verheij J., Brosnan M.J., Böcskei Z., Anstee Q.M., Bossuyt P.M. (2020). Enhanced liver fibrosis test for the non-invasive diagnosis of fibrosis in patients with NAFLD: A systematic review and meta-analysis. J. Hepatol..

