# Supplementary material for: FibroTest for Evaluating Fibrosis in Non-Alcoholic Fatty Liver Disease Patients: A Systematic Review and Meta-Analysis
Source: J Clin Med. 2021 May 29;10(11):2415. doi: 10.3390/jcm10112415 (PMC8198930; doi:10.3390/jcm10112415)
Supplement: Supplementary file 1 [file jcm-10-02415-s001.zip › jcm-1228996-supplementary.pdf]

supplementary material

Supplementary Table 1. PRISMA-DTA checklist

Section/topic	#	PRISMA-DTA Checklist Item	Reported on page #
**TITLE / ABSTRACT**			
Title	1	Identify the report as a systematic review (+/- meta-analysis) of diagnostic test accuracy (DTA) studies.	1
Abstract	2	Abstract: See PRISMA-DTA for abstracts.	1
**INTRODUCTION**			
Rationale	3	Describe the rationale for the review in the context of what is already known.	2
Clinical role of index test	D1	State the scientific and clinical background, including the intended use and clinical role of the index test, and if applicable, the rationale for minimally acceptable test accuracy (or minimum difference in accuracy for comparative design).	2
Objectives	4	Provide an explicit statement of question(s) being addressed in terms of participants, index test(s), and target condition(s).	2
**METHODS**			
Protocol and registration	5	Indicate if a review protocol exists, if and where it can be accessed (e.g., Web address), and, if available, provide registration information including registration number.	2
Eligibility criteria	6	Specify study characteristics (participants, setting, index test(s), reference standard(s), target condition(s), and study design) and report characteristics (e.g., years considered, language, publication status) used as criteria for eligibility, giving rationale.	3
Information sources	7	Describe all information sources (e.g., databases with dates of coverage, contact with study authors to identify additional studies) in the search and date last searched.	2-3
Search	8	Present full search strategies for all electronic databases and other sources searched, including any limits used, such that they could be repeated.	Supplementary Table 2
Study selection	9	State the process for selecting studies (i.e., screening, eligibility, included in systematic review, and, if applicable, included in the meta-analysis).	3
Data collection process	10	Describe method of data extraction from reports (e.g., piloted forms, independently, in duplicate) and any processes for obtaining and confirming data from investigators.	3
Definitions for data extraction	11	Provide definitions used in data extraction and classifications of target condition(s), index test(s), reference standard(s) and other characteristics (e.g. study design, clinical setting).	3

Risk of bias and applicability	12	Describe methods used for assessing risk of bias in individual studies and concerns regarding the applicability to the review question.	4
Diagnostic accuracy measures	13	State the principal diagnostic accuracy measure(s) reported (e.g. sensitivity, specificity) and state the unit of assessment (e.g. per-patient, per-lesion).	4
Synthesis of results	14	Describe methods of handling data, combining results of studies and describing variability between studies. This could include, but is not limited to: a) handling of multiple definitions of target condition. b) handling of multiple thresholds of test positivity, c) handling multiple index test readers, d) handling of indeterminate test results, e) grouping and comparing tests, f) handling of different reference standards	4
Section/topic	#	PRISMA-DTA Checklist Item	Reported on page #
Meta-analysis	D2	Report the statistical methods used for meta-analyses, if performed.	4
Additional analyses	16	Describe methods of additional analyses (e.g., sensitivity or subgroup analyses, meta-regression), if done, indicating which were pre-specified.	8
**RESULTS**			
Study selection	17	Provide numbers of studies screened, assessed for eligibility, included in the review (and included in meta-analysis, if applicable) with reasons for exclusions at each stage, ideally with a flow diagram.	5
Study characteristics	18	For each included study provide citations and present key characteristics including: a) participant characteristics (presentation, prior testing), b) clinical setting, c) study design, d) target condition definition, e) index test, f) reference standard, g) sample size, h) funding sources	5
Risk of bias and applicability	19	Present evaluation of risk of bias and concerns regarding applicability for each study.	5-6
Results of individual studies	20	For each analysis in each study (e.g. unique combination of index test, reference standard, and positivity threshold) report 2x2 data (TP, FP, FN, TN) with estimates of diagnostic accuracy and confidence intervals, ideally with a forest or receiver operator characteristic (ROC) plot.	6 and Supplementary figures
Synthesis of results	21	Describe test accuracy, including variability; if meta-analysis was done, include results and confidence intervals.	6
Additional analysis	23	Give results of additional analyses, if done (e.g., sensitivity or subgroup analyses, meta-regression; analysis of index test: failure rates, proportion of inconclusive results, adverse events).	6
**DISCUSSION**			
Summary of evidence	24	Summarize the main findings including the strength of evidence.	11

Limitations	25	Discuss limitations from included studies (e.g. risk of bias and concerns regarding applicability) and from the review process (e.g. incomplete retrieval of identified research).	12
Conclusions	26	Provide a general interpretation of the results in the context of other evidence. Discuss implications for future research and clinical practice (e.g. the intended use and clinical role of the index test).	12-13
**FUNDING**			
Funding	27	For the systematic review, describe the sources of funding and other support and the role of the funders.	13

Supplementary Table 2. Preliminary search strategy for Medline

1	exp Fatty Liver/
2	(NAFL* or NASH*).mp.
3	"non-alcoholic fatty liver disease*".mp.
4	(((fatty or fat or steato*) adj3 (liver* or hepat*)) or steatohepat* or (visceral adj2 steato*)).ti,ab.
5	1 or 2 or 3 or 4
6	exp "sensitivity and specificity"/ or exp "mass screening"/ or "reference values"/ or "false positive reactions"/ or "false negative reactions"/ or specificit$.tw. or screening.tw. or false positive$.tw. or false negative$.tw. or accuracy.tw. or predictive value$.tw. or reference value$.tw. or roc$.tw. or likelihood ratio$.tw. or predictive value$.tw.
7	exp BIOMARKERS/
8	(biomarker$ or marker$).ti,ab,kf,rn.
9	(test* or measure* or level* or diagnos* or ratio or score*).ti,ab.
10	((biomarker* or marker*) adj4 (test* or measure* or level* or ratio or score*)).ti,ab.
11	Laboratory Test$.ti,ab,kf.
12	(Cytokeratin-18 or Keratin-18 or CYK18 or CYK-18 or KRT18 or KRT-18).ti,ab,kf.
13	exp cytokeratin 18/
14	((Spectroscopy or LS23 or spectrometer) and DiaFir).ti,ab,kw. or MIR-FEWS.ti,ab,kf.
15	("enhanced liver fibrosis" or ELFscore or ELFtest).ti,ab,kf.
16	(Glycomics-based or Glyco-Liver or N-glycans or Nglycans or (Glyco* adj3 (profile or test or measure))).ti,ab,kf.
17	((SOMAscan or SOMAmers) adj4 (V4 or plex)).mp.
18	(miR-122 or miR-34a or (miR122 or miR34a) or (micro RNA 122 or micro RNA 34a)).ti,ab,kf.
19	(Alpha-2 Macroglobulin or A2M or Alpha2 Macroglobulin or Alpha2-Macroglobulin or A2 Macroglobulin or A2-macroglobulin or a2-macroglobulin or a2 macroglobulin or a2macroglobulin).ti,ab,kf.
20	(haptoglobin or HP or Hpt or a2-glycoprotein).ti,ab,kf.
21	(apolipoprotein a1 or APOA1 or Apolipoprotein A-I or apoA-I or apo A-I or apoA-1 or apo A-1 or apo-AI or Apo-A1).ti,ab,kf.
22	(((OWLiver or OWL) adj2 test*) or (OWL adj2 metabolomic*)).ti,ab,kf.
23	(type III pro-collagen or type III procollagen or type 3 pro-collagen or type 3 procollagen or Pro-C3 or Proc3).ti,ab,kf.
24	((7S domain adj3 collagen type IV) or P4NP_7S or P4NP7S or P4NP-7S).ti,ab,kf.
25	((((A2 or A9) adj3 (fibrogenesis or fibrolysis)) or ((fibrogenesis or fibrolysis) adj3 marker*)) and (A2 or A9)).ti,ab,kf.
26	(((extracellular matrix or matricellular or ECM) adj2 molecules) and (A2 or A9)).ti,ab,kf.

27	(type VI pro-collagen or type VI procollagen or type 6 pro-collagen or type 6 procollagen or Pro-C6 or Pro C6 or Proc6).ti,ab,kf.
28	((nafld or bard or ferritin* or fibrosis) adj4 (test* or measure* or level* or ratio or score*)).ti,ab,kf.
29	FIB-4.ti,ab.
30	((glutamic-pyruvic transaminase or glutamic-oxaloacetic transaminase or sgot or sgpt or alt or ast) adj4 (test* or measure* or level* or ratio or score*)).ti,ab.
31	(alanine adj2 (aminotransferase or transaminase) adj4 (test* or measure* or level* or ratio or score*)).ti,ab.
32	((ast-to-platelet ratio index or apri or elf or enhanced liver fibrosis or nash) adj4 (panel or test* or measure* or level* or score*)).ti,ab.
33	((Aspartate or AST or Aminotransferase) adj3 Platelet adj2 ratio adj2 index).ti,ab.
34	(APRI or APR-index or APRindex or ("AST/platelet" adj3 "ratio index")).ti,ab.
35	(fibro-test* or Fibrotest* or fibrosure* or fibro-sure* or fibrometer or fibro-meter* or fib4 or fib-4).ti,ab,kf.
36	Hepascore.mp.
37	(fibroblast activation protein* or FAP).mp.
38	((Apolipoprotein adj3 F) or Apo-F or ApoF or Apo F).mp.
39	7 or 8 or 9 or 10 or 11 or 12 or 13 or 14 or 15 or 16 or 19 or 20 or 21 or 22 or 23 or 24 or 25 or 26 or 27 or 28 or 29 or 30 or 31 or 32 or 33 or 34 or 35 or 36 or 37 or 38
40	5 and 6 and 39
41	exp animals/ not humans/
42	40 not 41

*In our initial search we looked for several non-invasive blood derived biomarkers and in the updates we only searched for FibroTest based diagnostic accuracy studies.

ActiTest, another test from Biopredictive Paris, which includes the same components plus alanine-aminotransferase (ALT), is used for assessment of necroinflammatory activity; this test, however, was not included in this systematic review [1].

Supplementary Table 3: Conversion grid for the stages of liver fibrosis

Fibrosis Distribution	NASH CRN fibrosis stage[2]	Ishak Fibrosis stage[3]	METAVIR fibrosis stage[4]	Knodell Fibrosis stage[5]	Scheuer Fibrosis stage[6]	International Association for Study of the Liver (IASL) scoring system[7]	The Batts-Ludwig system [8]	EPoS staging system[9]
No excess fibrosis	F0	F0	F0	F0	F0	F0	F0	F0
Portal or perisinusoidal	F1	F1	F1	F1	F1	F1	F1	F1
Portal and perisinusoidal	F2	F2	F1	F1	F1	F1	F2	F2
Bridging	F3	F3-4	F2-3	F3	F2-3	F2-3	F3	F3-4
Nodules	F4	F5- 6	F4	F4	F4	F4	F4	F5- 6

Supplementary Table 4: Histological scoring systems developed to characterize changes in NAFLD progression

**Brunt criteria (Necroinflammatory Grading System for Steatohepatitis)[10]**	A system for semiquantitative evaluation for the unique lesions recognized for NASH, developed for NASH and not to encompass the entire spectrum of NAFLD	
	Mild, grade 1	Steatosis (predominantly macrovesicular) involving up to 66% of biopsy; may see occasional ballooned zone 3 hepatocytes; scattered rate intra-acinar pmn's 6 intraacinar lymphocytes; no or mild portal chronic inflammation.
	Moderate, grade 2	Steatosis of any degree; ballooning of hepatocytes (predominantly zone 3) obvious; intra-acinar pmn's noted, may be associated with zone 3 pericellular fibrosis; portal and intra-acinar chronic inflammation noted, mild to moderate
	Severe, grade 3	Panacinar steatosis; ballooning and disarray obvious, predominantly in zone 3; intra-acinar inflammation noted as scattered pmn's, pms's associated with ballooned hepatocytes 6 mild chronic inflammation; portal chronic inflammation mild or moderate, not marked.
**Matteoni criteria (The original criteria for NAFLD subtypes)[11]**	Developed to encompass the entire spectrum of NAFLD. NAFLD types 3 and 4 were considered to be NASH	
	NAFLD type 1	Steatosis alone
	NAFLD type 2	Steatosis with lobular inflammation only
	NAFLD type 3	Steatosis with hepatocellular ballooning
	NAFLD type 4	Steatosis with Mallory-Denk bodies or fibrosis
**NAFLD Activity Score (NAS) [2]**	A semi-quantitative scoring system for assessing the range of histological features of NAFLD. Comprised of 3 histological features: steatosis (0-3), lobular inflammation (0-3), hepatocellular ballooning (0-2)	
	Steatosis	
	Lobular inflammation	
	Hepatocellular ballooning	
	Fibrosis	
**SAF score (steatosis, activity, fibrosis) [12]**	SAF score dissociates grade of steatosis, grade of activity, and stage of fibrosis	
	The steatosis score (S)	From 0 to 3 (S0: <5%; S1: 5%-33%, mild; S2: 34%-66%, moderate; S3: >67%, marked).
	Activity grade (A)	From 0-4, is the unweighted addition of hepatocyte ballooning (0-2) and lobular inflammation (0-2)<br>A0 (A ¼ 0) no activity, A1 (A ¼ 1), mild activity, A2 (A ¼ 2), moderate activity, A3 severe activity.
	Stage of fibrosis (F)	stage 0 (F0) (none); stage 1 (F1): 1a or 1b perisinusoidal zone 3 or 1c portal fibrosis, stage 2 (F2): perisinusoidal and periportal fibrosis without bridging, stage 3 (F3): bridging fibrosis and stage 4 (F4): cirrhosis
**Younossi criteria[13]**	1	Any degree of steatosis along with centrilobular ballooning and/or Mallory- Denk bodies

	2	Any degree of steatosis along with centrilobular pericellular/perisinusoidal fibrosis or bridging fibrosis in the absence of another identifiable cause

Supplementary Table 5. Number of excluded papers for each exclusion reason

Reason For Exclusion/ All Biomarkers	***Number Of Studies***
**No Biopsy**	9
**Wrong Population**	11
**Wrong Study Design**	5
**Wrong Biomarker**	13
**Wrong Outcome**	4
**No Full Text Available**	12
**Not Relevant Abstracts**	9
**Reason For Exclusion/ Fibrotest Full Texts**	***Number Of Studies***
**No Biopsy**	1
**Wrong Population**	2
**Abstract of Included Full Text**	2
**Wrong Outcome**	1

Supplementary Table 6. Correspondence between the NASH CRN and the METAVIR systems reason. Reported by Boursier et al. 2017[14]

<table>
<tr><th>Metavir</th><th>NASH CRN</th><th colspan="2">Present study (Boursier et al. 2017)</th></tr>
<tr><td>F0</td><td>F0 or F1 (isolated perisinusoidal fibrosis)</td><td colspan="2" rowspan="2">No/mild fibrosis</td></tr>
<tr><td>F1</td><td>F1 (isolated periportal fibrosis) or F2</td></tr>
<tr><td>F2</td><td>F3</td><td rowspan="2">Septal fibrosis</td><td rowspan="3">Advanced fibrosis</td></tr>
<tr><td>F3</td><td>F3</td></tr>
<tr><td>F4</td><td>F4</td><td>Cirrhosis</td></tr>
</table>

Supplementary Table 7. Biopsy criteria of studies included in the meta-analysis

Study Id	Number of cases	Age, Y (SD)	Biopsy samples						
			**Needle gauge (mm)**	**Biopsy Length (mm)**	**Portal tracts**	**Time interval**	**Pathologist**	**Blind to other tests**	**Details**
**Bril 2020**	162	57 (8.5)	NR	17	9	NR	Single expert	Yes	NR
**Boursier 2019**	938	56.5 (12.1)	NR	27 (12)	NR	< 1 week	Single expert hepatopathologist in each center	Yes	NR
**Bril 2019**	220	58 (9)	NR	NR	NR	NR	NR	NR	NR
**Poynard 2012**	494	42.2 (11.3)	NR	13.8 (10.8)	NR	<6 months	Centralized pathologists	Yes	Biopsies were routinely stained with hematoxylin-eosin and Masson's trichrome.
**Adams 2011**	242	46.8 (12.4)	NR	16 (6-50)	NR	At the same time	Single histopathologist	Yes	NR
**Sebastiani 2011**	190	51.2 (13.3)	NR	17.7 (8.4)	10.6 (5.9)	Same day	Single pathologist	Yes	NR
**Ratziu 2006**	267	51.2 (0.9)∞	NR	19.2	15.3	< 3 months	Single pathologist	Yes	Liver biopsies were fixed, paraffin-embedded, and stained with at least hematoxylin-eosin-safran, iron staining, and Masson's trichrome or picrosirius red for collagen

Supplementary Table 8. METAVIR scoring systems and pre-determined FibroTest cutoffs

SAF scoring system		METAVIR scoring system	
**Classes**	**Definition**	**Definition**	**Recommended cutoffs**
**Fibrosis**			**FibroTest**
F0	None	None	0.00
F1	Perisinusoidal or portal	Portal fibrosis	0.27
F2	Perisinusoidal and portal without bridging	Few septa	0.48
F3	Bridging	Many septa	0.58
F4	Cirrhosis	Cirrhosis	0.74

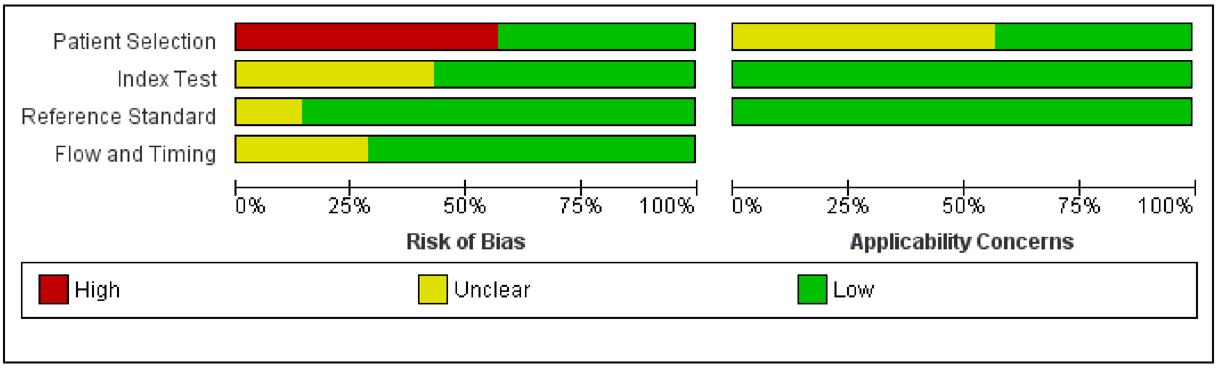

Supplementary Fig.1. Graphical summary of the methodological quality of included studies using the QUADAS-2 tool

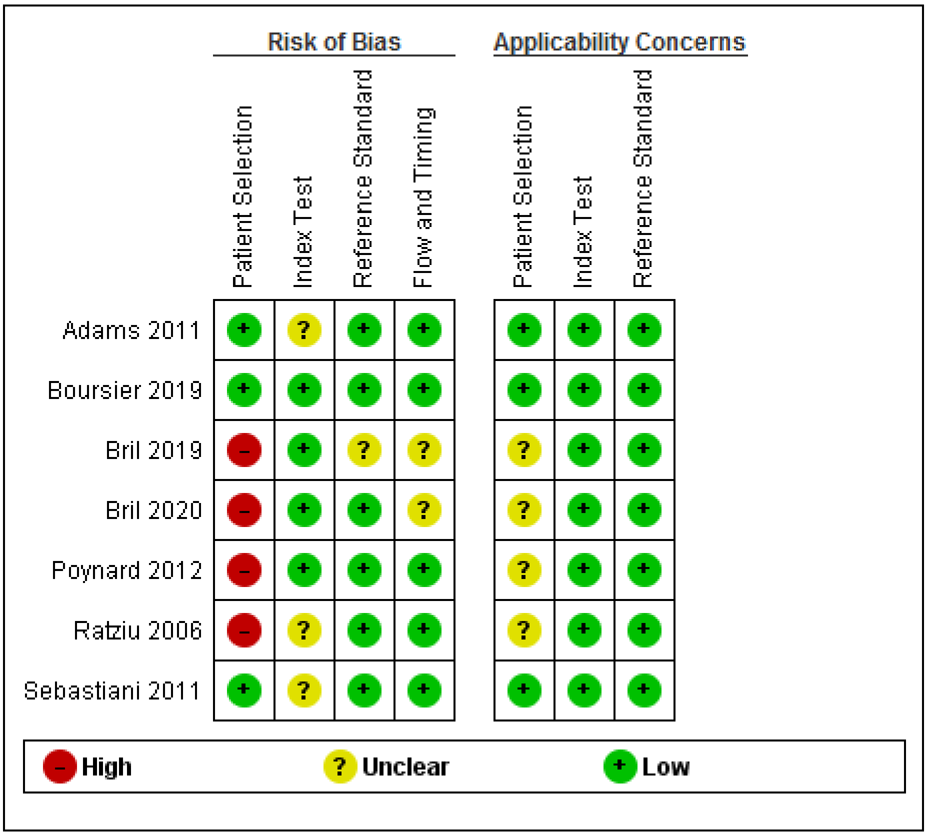

Supplementary Fig.2. Methodological quality of each of the included studies per domain of the QUADAS-2 tool

Study	TP	FP	FN	TN	Thresholds	Sensitivity (95% CI)	Specificity (95% CI)
Adams 2011	32	19	21	170	0.47	0.60 [0.46, 0.74]	0.90 [0.85, 0.94]
Boursier 2019a	318	273	65	282	0.3	0.83 [0.79, 0.87]	0.51 [0.47, 0.55]
Boursier 2019b	174	86	209	469	0.58	0.45 [0.40, 0.51]	0.85 [0.81, 0.87]
Boursier 2019c	118	47	265	508	0.7	0.31 [0.26, 0.36]	0.92 [0.89, 0.94]
Bril 2020	20	34	11	97	0.35	0.65 [0.45, 0.81]	0.74 [0.66, 0.81]
Poynard 2012	4	2	45	443	0.48	0.08 [0.02, 0.20]	1.00 [0.98, 1.00]
Ratziu 2006a	32	67	3	165	0.3	0.91 [0.77, 0.98]	0.71 [0.65, 0.77]
Ratziu 2006b	9	7	26	225	0.7	0.26 [0.12, 0.43]	0.97 [0.94, 0.99]

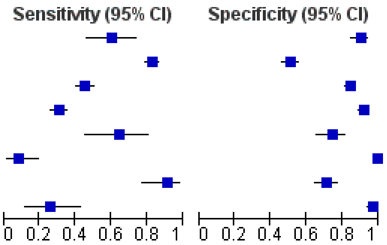

Supplementary Fig.3. Forest plot of all included studies for advanced fibrosis. FN = false negative; FP = false positive; TN = true negative; TP = true positive. The forest plot shows an estimate of sensitivity and specificity from each study and the threshold used. The horizontal lines around each box depict the confidence intervals. Studies with more than one threshold are labelled with letters

Study	TP	FP	FN	TN	Threshold	Sensitivity (95% CI)	Specificity (95% CI)
Adams 2011	57	39	40	106	0.57	0.59 [0.48, 0.69]	0.73 [0.65, 0.80]
Boursier 2019	312	61	323	242	0.74	0.49 [0.45, 0.53]	0.80 [0.75, 0.84]
Bril 2019	27	26	22	76	0.35	0.55 [0.40, 0.69]	0.75 [0.65, 0.83]
Ratziu 2006	56	45	17	149	0.3	0.77 [0.65, 0.86]	0.77 [0.70, 0.83]
Sebastiani 2011	40	22	58	70	0.75	0.41 [0.31, 0.51]	0.76 [0.66, 0.84]

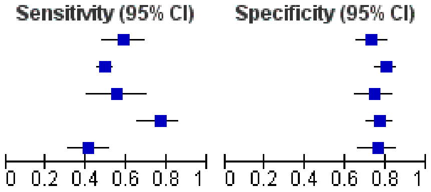

Supplementary Fig.4. Forest plot of studies investigating diagnostic accuracy of FibroTest for detecting significant fibrosis. FN = false negative; FP = false positive; TN = true negative; TP = true positive. The forest plot shows an estimate of sensitivity and specificity from each study and the threshold used. The horizontal lines around each box depict the confidence intervals.

Study	TP	FP	FN	TN	Threshold	Sensitivity (95% CI)	Specificity (95% CI)
Adams 2011	18	17	7	200	0.57	0.72 [0.51, 0.88]	0.92 [0.88, 0.95]
Boursier 2019	51	81	75	731	0.74	0.40 [0.32, 0.50]	0.90 [0.88, 0.92]
Sebastiani 2011	20	9	6	155	0.75	0.77 [0.56, 0.91]	0.95 [0.90, 0.97]

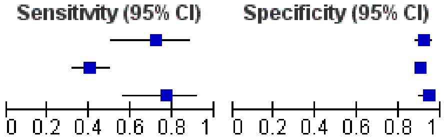

Supplementary Fig.5. Forest plot of studies investigating diagnostic accuracy of FibroTest for detecting cirrhosis. FN = false negative; FP = false positive; TN = true negative; TP = true positive. The forest plot shows an estimate of sensitivity and specificity from each study and the threshold used. The horizontal lines around each box depict the confidence intervals.

Study	TP	FP	FN	TN	Threshold	Sensitivity (95% CI)	Specificity (95% CI)
Boursier 2019	603	36	246	53	0.74	0.71 [0.68, 0.74]	0.60 [0.49, 0.70]
Bril 2019	64	14	49	24	0.26	0.57 [0.47, 0.66]	0.63 [0.46, 0.78]
Poynard 2012	34	6	218	236	0.27	0.13 [0.10, 0.18]	0.98 [0.95, 0.99]

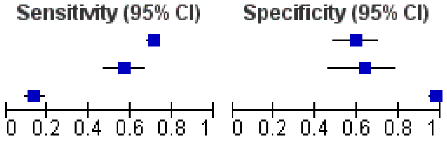

Supplementary Fig.6. Forest plots of studies investigating diagnostic accuracy of FibroTest for detecting any fibrosis. FN = false negative; FP = false positive; TN = true negative; TP = true positive. The forest plot shows an estimate of sensitivity and specificity from each study and the threshold used. The horizontal lines around each box depict the confidence intervals.

References

1. Poynard, T., et al., *FibroTest-FibroSURE™: towards a universal biomarker of liver fibrosis?* Expert review of molecular diagnostics, 2005. **5**(1): p. 15-21.
2. Kleiner, D.E., et al., *Design and validation of a histological scoring system for nonalcoholic fatty liver disease.* Hepatology, 2005. **41**(6): p. 1313-21.
3. Ishak, K., et al., *Histological grading and staging of chronic hepatitis.* J Hepatol, 1995. **22**(6): p. 696-9.
4. *Intraobserver and interobserver variations in liver biopsy interpretation in patients with chronic hepatitis C. The French METAVIR Cooperative Study Group.* Hepatology, 1994. **20**(1 Pt 1): p. 15-20.
5. Knodell, R.G., et al., *Formulation and application of a numerical scoring system for assessing histological activity in asymptomatic chronic active hepatitis.* Hepatology, 1981. **1**(5): p. 431-5.
6. Scheuer, P.J., *Classification of chronic viral hepatitis: a need for reassessment.* Journal of hepatology, 1991. **13**(3): p. 372-374.
7. Desmet, V.J., et al., *Classification of chronic hepatitis: diagnosis, grading and staging.* Hepatology, 1994. **19**(6): p. 1513-1520.
8. Batts, K.P. and J. Ludwig, *Chronic hepatitis. An update on terminology and reporting.* The American journal of surgical pathology, 1995. **19**(12): p. 1409-1417.
9. Bedossa, P., et al., *The EPoS staging system is a reproducible 7-tierfibrosis score for NAFLD adapted both to glass slides and digitized images (e-slides).* Journal of Hepatology, 2018. **68**: p. S553.
10. Brunt, E.M., et al., *Nonalcoholic steatohepatitis: a proposal for grading and staging the histological lesions.* The American journal of gastroenterology, 1999. **94**(9): p. 2467.
11. Matteoni, C.A., et al., *Nonalcoholic fatty liver disease: a spectrum of clinical and pathological severity.* Gastroenterology, 1999. **116**(6): p. 1413-1419.
12. Bedossa, P., et al., *Histopathological algorithm and scoring system for evaluation of liver lesions in morbidly obese patients.* Hepatology, 2012. **56**(5): p. 1751-9.
13. Younossi, Z.M., et al., *Pathologic criteria for nonalcoholic steatohepatitis: interprotocol agreement and ability to predict liver-related mortality.* Hepatology, 2011. **53**(6): p. 1874-1882.
14. Boursier, J., et al., *A stepwise algorithm using an at-a-glance first-line test for the non-invasive diagnosis of advanced liver fibrosis and cirrhosis.* Journal of hepatology, 2017. **66**(6): p. 1158-1165.
